# An Elevated Maternal Plasma Soluble fms-Like Tyrosine Kinase-1 to Placental Growth Factor Ratio at Midtrimester Is a Useful Predictor for Preeclampsia

**DOI:** 10.1155/2013/202346

**Published:** 2013-12-04

**Authors:** Mahmoud Fathy Hassan, Nancy Mohamed Ali Rund, Ahmed Husseiny Salama

**Affiliations:** ^1^Faculty of Medicine, Ain Shams University, Egypt; ^2^King Abdulaziz Airbase Hospital, P.O. Box 570, Dhahran 31932, Saudi Arabia

## Abstract

*Background.* To assess the ability of mid-trimester sFlt-1/PlGF ratio for prediction of preeclampsia in two different Arabic populations. *Methods.* This study measured levels of sFlt-1, PlGF, and sFlt-1/PlGF ratio at midtrimester in 83 patients who developed preeclampsia with contemporary 250 matched controls. *Results.* Women subsequently developed preeclampsia had significantly lower PlGF levels and higher sFlt-1 and sFlt-1/PlGF ratio levels than women with normal pregnancies (*P* < 0.0001 for all). Women who with preterm preeclampsia had significantly higher sFlt-1 and sFlt-1/PlGF ratio than term preeclamptic women (*P* = 0.01, 0.003, resp.). A cutoff value of 3198 pg/mL for sFlt-1 was able to predict preeclampsia with sensitivity, specificity, and accuracy of 88%, 83.6%, and 84.7%, respectively, with odds ratio (OR) 37.2 [95% confidence interval (CI) 17.7–78.1]. PIGF at cutoff value of 138 pg/mL was able to predict preeclampsia with sensitivity, specificity, and accuracy of 85.5%, 77.2%, and 79.3%, respectively, with OR 20 [95% CI, 10.2–39.5]. The sFlt-1/PIGF ratio at cutoff value of 24.5 was able to predict preeclampsia with sensitivity, specificity, and accuracy of 91.6%, 86.4%, and 87.7%, respectively with OR 67 [95% CI, 29.3–162.1]. *Conclusion.* Midtrimester sFlt-1/PlGF ratio displayed the highest sensitivity, specificity, accuracy, and OR for prediction of preeclampsia, demonstrating that it may stipulate more effective prediction of preeclampsia development than individual factor assay.

## 1. Introduction

Preeclampsia affects nearly five percent of pregnancies, and it is characterized by hypertension and proteinuria after 20 weeks of gestation and results in maternal and fetal morbidity and mortality during pregnancy [1, 2]. Forty-two percent of all maternal deaths per year and 15% of all preterm deliveries are correlated to preeclampsia [3]. Despite intense research efforts, the pathogenesis of preeclampsia is still mysterious, but it is mostly multifactorial [4].

The accumulation of evidence suggests that preeclampsia results from the imbalance between placental angiogenic and antiangiogenic factors that harm maternal vascular endothelium, resulting in clinical features for this condition [5–9]. Because of the high prevalence and seriousness of preeclampsia, evaluation of various angiogenic and antiangiogenic factors in both serum and plasma have been tested as diagnostic markers for preeclampsia along with an assessment for the probability of their use in prediction of preeclampsia development [6, 10–13]. Elevated serum levels of the antiangiogenic soluble fms-like tyrosine kinase-1 (sFlt-1) are found involved in preeclampsia [5, 14–16]. A pathological role for the antiangiogenic factor sFlt-1 has been established in pregnant animals [5, 17]. A preeclampsia-like syndrome has been reported in pregnant rats that have been given adenovirus expressing sFlt-1 [5]. In humans, serum levels of the angiogenic placental growth factor (PIGF) are reduced in women with preeclampsia [18–20]. An increase in sFlt-1 and a decrease in PlGF have been revealed in maternal serum five to 10 weeks before the onset of preeclampsia [6]. It has been postulated that these changes in sFlt-1 and PlGF may contribute to the pathogenesis of preeclampsia [6, 21]. Thus, these two particular factors have been proposed as candidates for an efficient screening in the prediction of preeclampsia [12, 22, 23].

Formerly, many studies assessed sFlt-1 and PlGF for the prediction of preeclampsia development. However, few studies reported the sFlt-1/PlGF ratio in the prediction of preeclampsia. In the present study, we created a multicenter study to investigate the ability of midtrimester serum levels of sFlt-1, PlGF, and sFlt-1/PlGF ratio to identify women at high risk for development of preeclampsia and to determine whether the use of sFlt-1/PlGF ratio was superior to the use of individual biomarkers sFlt-1 and PlGF in the prediction of preeclampsia.

## 2. Patients and Methods

We conducted a nested case-control study within a cohort of 734 women with a singleton pregnancy. Women who developed subsequently preeclampsia (*n* = 83) made up the case group, while the control group consisted of randomly selected women with normal pregnancy outcome (*n* = 250). The study was conducted as a multicenter study at 3 large medical centers (two at different regions in Saudi Arabia and one in Egypt) in the period between October 2009 and March 2012. An identical study protocol and data collection form was used at each center. The local Research Ethics Committee and Institutional Review Boards approved the procedure and all participants gave an informed written consent before involvement in the study. Sample collection was taken during the routine antenatal screening (at 15–20 weeks of gestation). Background data of the patients were provided by the study centers in the form of a completed case report form.

Inclusion criteria for the cohort were the presence of informed consent, maternal age >16 years, a normal pregnancy outcome in the control group, and the presence of preeclampsia as stated by the definitions specified below in the case group. Exclusion criteria were multiple gestation, antiphospholipid antibody syndrome, systemic lupus erythematosus, or any other autoimmune disease as well as chronic corticosteroid drug use. A normal pregnancy outcome denoted a mother who delivered a healthy baby at term (37 weeks of gestation or more) without medical or obstetric complications such as diabetes, chronic hypertension, renal insufficiency, congenital anomalies, or fetal demise. Women in the control group were normotensive, normoglycemic and had no proteinuria during pregnancy. The diagnosis of preeclampsia was based on the modified American College of Obstetricians and Gynecologists criteria [24]. Preeclampsia was defined as a blood pressure ≥140/90 mm Hg on two occasions: 2 hours to 2 weeks apart after 20 weeks of gestation and proteinuria of ≥300 mg/24 hour after 20 weeks of gestation. Proteinuria on presentation was defined as urine dipstick with 2+ protein or more or 24-hour urine protein ≥300 mg/day. Preterm delivery was defined as the delivery of a neonate before completing 37 weeks of gestation.

Maternal blood samples were obtained from all participants at 15 to 20 weeks of gestation during the routine antenatal screening. Maternal plasma samples were isolated by 2,500 g centrifugation for 10 minutes. Aliquots of maternal plasma were stored at −80°C for future analysis. Levels for sFlt-1 and PlGF were measured with a commercially available enzyme-linked immunosorbent assay (ELISA) (ELISA, R&D System, Minneapolis, MN, USA) according to the manufacturer's instructions. All samples were run in duplicate on the assay plate. If more than 10% variation existed between duplicates, the assay was repeated and the average was reported. Test of sensitivity for the assay was 13.3 and 7 pg/mL for sFlt-1 and PlGF, respectively. The limit of detection was 31.2 pg/mL for sFlt-1 and 15.6 pg/mL for PlGF. The interassay and intra-assay coefficients of variation were 5.4% and 11.2% for sFlt-1, 5.1% and 12.1% for PlGF. The average of duplicate assays represented the value of individual samples in the statistical analysis. The sFlt-1/PlGF ratio was calculated as the proportion between both values. Personnel who performed the assays of analytes were blinded to the clinical information.

### 2.1. Statistical Analyses

Shapiro-Wilk normality test was used to test the data for a normal distribution. Continuous variables with normal distribution were compared by unpaired *t*-test, and data were presented as mean ± standard deviation. Nonnormally distributed variables were analyzed by the Mann-Whitney *U* test, and data were presented as median and interquartile range. Fisher's exact test was used for comparisons between categorical variables, and data were presented as number and percentage. In all tests, a two-tailed *P* value less than 0.05 was considered statistically significant.

To assess the clinical utility of individual factor assay and the sFlt-1/PlGF ratio in the prediction of preeclampsia, we used receiver operating characteristics (ROC) curve analysis to assess the optimal cutoff value of each factor. The optimal cutoffs of the analytes sFlt-1, PlGF, and the sFlt-1/PlGF ratio were set at the best accuracy value based on the ROC curve analysis. The sensitivity, specificity, positive and negative predictive values, positive and negative likelihood ratio, and accuracy for the optimal cutoff value were calculated. The odds ratio (OR) with a 95% confidence interval (CI) was calculated to consider the predictive competence of the selected cutoff values. A false positive rate (calculated as 100 − specificity) at 5, 10, and 15% sensitivity was compared between each factor and the sFlt-1/PlGF ratio. Statistical analysis was performed with the Statistical Package for Social Sciences 14.0 (SPSS Inc., Chicago, IL, USA), GraphPad Prism version 6, and Microsoft Excel 2003.

### 2.2. Sample Size Justification

Estimation of sample size was made by a priori analysis using the G*power3 program (Heinrich-Heine-Universität, Düsseldorf, Germany), with a wanted power of 95% and an *α* error of 0.05 to detect a difference of approximately 30% between the case and the control groups provided that the primary outcome measure was the sFlt-1/PlGF ratio (estimated from a previous observation of sFlt-1/PlGF ratio in preeclampsia [25]). This estimated a sample size of 74 participants from the case group and 220 participants from the control group (ratio 1 : 3).

## 3. Results

During the study period between October 2009 and March 2012, a cohort of 734 women with singleton pregnancies were enrolled. 71 women did not complete the followup. One hundred fifty-three women did not meet the criteria of the study during analysis of the data, while 16 patients were excluded from analysis due to incomplete records. Eighty-three women who ultimately developed preeclampsia were compared to a randomly selected 250 women (out of 411 women with normal pregnancy outcome) using random sampling with SPSS14.0 software.

The clinical characteristics of the study population were shown in Table 1. No significant difference in women ages has been shown in both groups. Sixty-six (79.5%) participants in the case group and 178 (70.4%) participants in the control group were nulliparous with no statistical difference. At blood sampling, gestational age, body mass index, systolic blood pressure, and diastolic blood pressure were not significantly different between the two groups. In contrast, gestational age at delivery and birth weight were significantly lower for participants in the case group than for those in the control group (both *P* < 0.0001).

**Figure 1:**
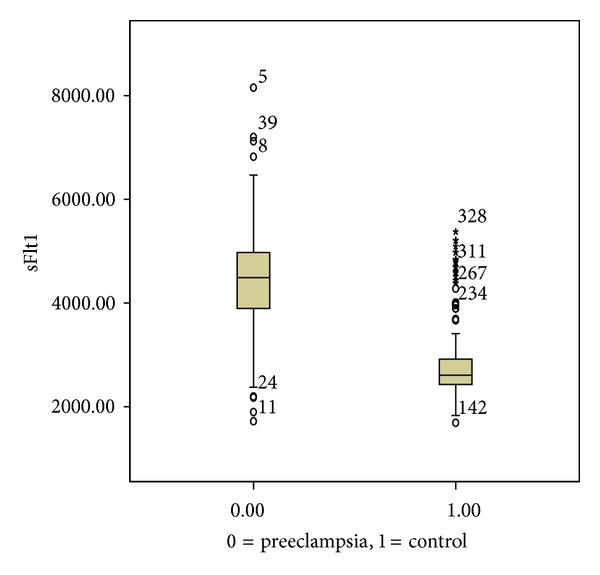
Median and interquartile range of fms-like tyrosine kinase (sFlt-1) in case group, 4490 (961) and in control group, 2591 (400); *P* < 0.0001.

Maternal plasma concentrations of sFlt-1, PlGF, and sFlt-1/PlGF ratio in both groups were displayed in Table 2. Median (interquartile range) plasma level of sFlt-1 was significantly higher in the case group compared to women in the control group (4490 (1080) versus 2590 (419), resp., *P* < 0.0001) (Figure 1).

**Figure 2:**
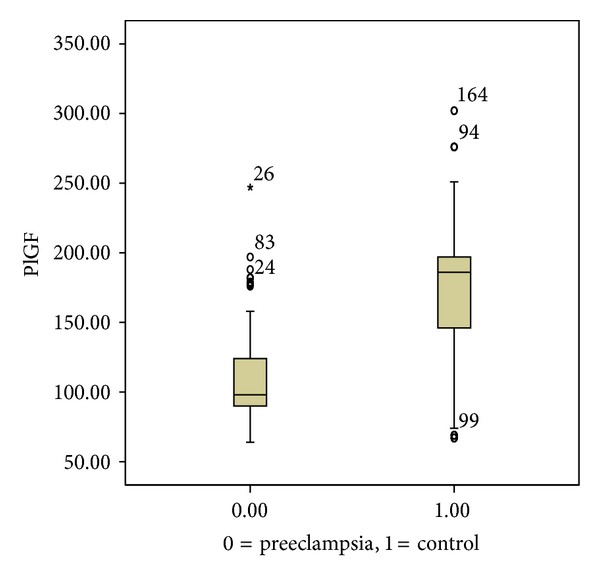
Median and interquartile range of placental growth factor in case group, 97 (29) and in control group, 186 (57.5); *P* < 0.0001.

In contrast, median plasma level of maternal PlGF was significantly lower in women of the case group compared to those in the control group (98 (35) versus 186 (52), resp., *P* < 0.0001) (Figure 2).

**Figure 3:**
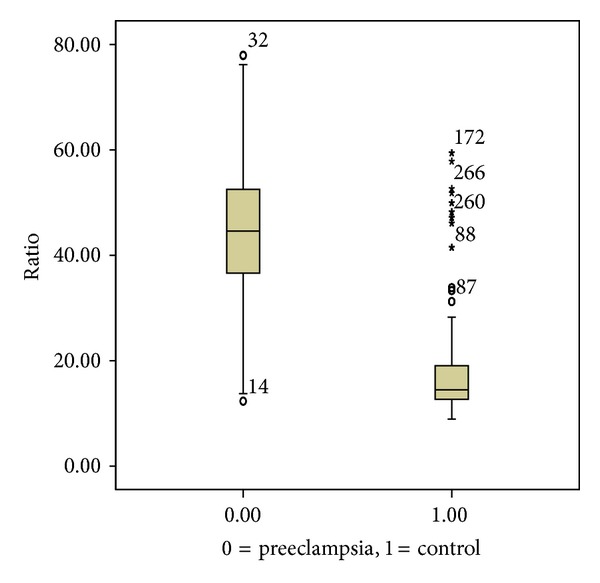
Median and interquartile range of sFlt-1/PlGF ratio in case group, 44.8 (16.2) and in control group, 14.4 (6.4); *P* < 0.0001.

In women with preeclampsia, the median sFlt-1/PlGF ratio was significantly higher compared to the control group (44.8 (16.2) versus 14.4 (6.1), resp., *P* < 0.0001) (Figure 3).

In Table 3, serum levels of sFlt-1, PlGF, and sFlt-1/PlGF ratio were compared according to the gestational age at delivery (term and preterm preeclampsia). Serum sFlt-1 and sFlt-1/PlGF ratio were significantly higher in the preterm-delivery group than in women with term delivery (*P* = 0.01 and 0.003, resp.). On the contrary, serum level of PlGF showed no significant difference between patients who had term or preterm preeclampsia.

**Figure 4:**
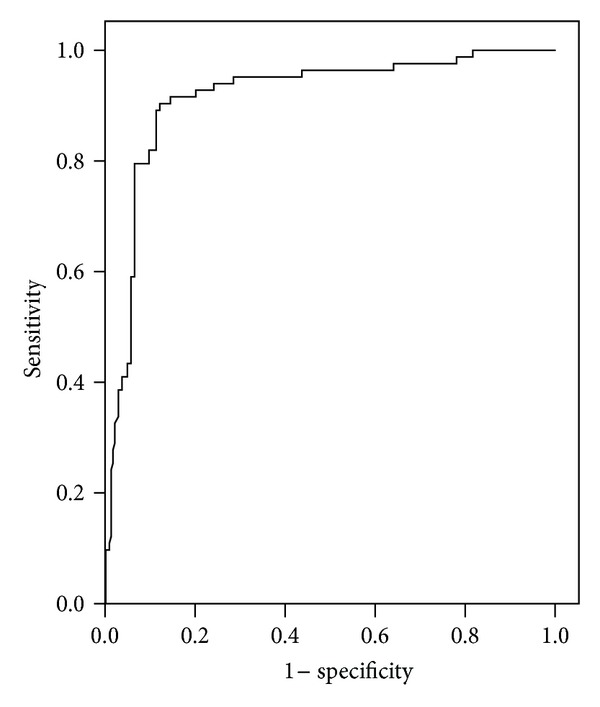
Receiver operating characteristic (ROC) curve of fms-like tyrosine kinase (sFlt-1) in the prediction of preeclampsia. Area under the curve (AUC) = 0.891 (95% CI: 0.835–0.947; *P* < 0.0001).

**Figure 5:**
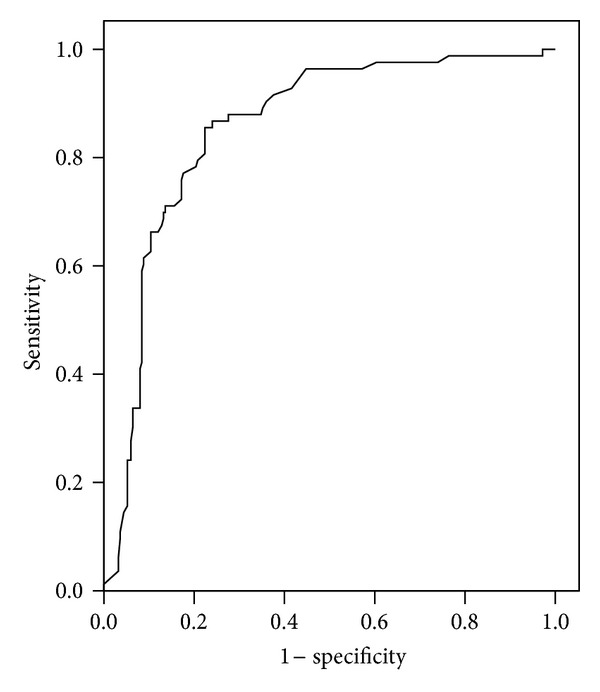
Receiver operating characteristic (ROC) curve of placental growth factor (PlGF) in the prediction of preeclampsia. Area under the curve (AUC) = 0.863 (95% CI: 0.818 to 0.907; *P* < 0.0001).

Area under the curve from receiver operating characteristic (ROC) for each marker was shown in Table 4. ROC curves analyses of sFlt-1 and PlGF as individual markers demonstrated convincing evidence for the use of these markers in identifying women at risk for developing preeclampsia with areas under the curve equal to 0.875 and 0.855 for sFlt-1 and PlGF, respectively (Figures 4 and 5).

**Figure 6:**
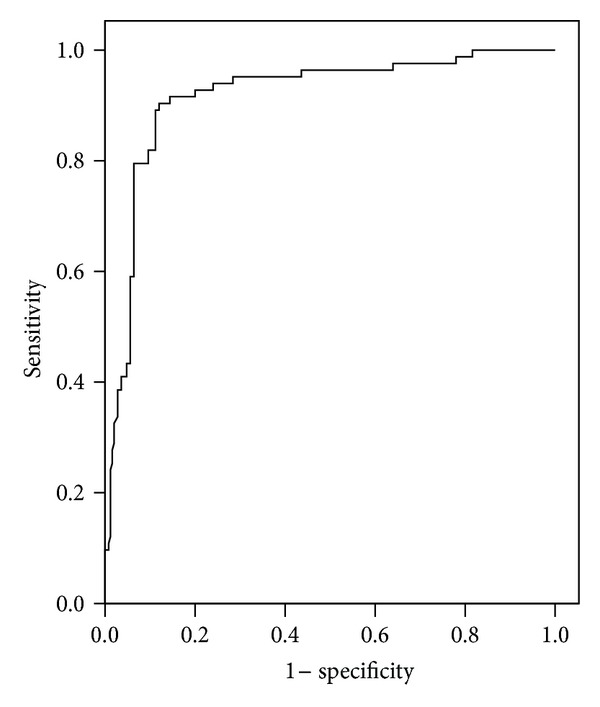
Receiver operating characteristic (ROC) curve of sFlt-1/PlGF ratio in the prediction of preeclampsia. Area under the curve (AUC) = 0.916 (95% CI: 0.877 to 0.955; *P* < 0.0001).

But the predictive accuracy of sFlt-1/PlGF ratio had superior performance to other parameters measured as demarcated by greater AUC (0.917) (Figure 6).

Moreover, sFlt-1/PlGF ratio had a higher sensitivity for preeclampsia at false positive rates of 5%, 10%, and 15% (59%, 81.9%, and 91.6%, resp.) than individual factor (Table 4).

Table 5 displayed the cutoff value of each factor at the best overall accuracy based on the ROC curve analysis with estimation of odds ratio (OD) and 95% confidence interval (CI) at that value. The resulting odds ratios showed that the risk of subsequent preeclampsia development was elevated in women with levels higher than the cutoff value compared with women whose values were less than the cutoff value with odds ratio (95% CI) 37.2 [7.7–78.1] for sFlt-1 and 67 [29.3–162.1] for the sFlt-1/PlGF ratio. On the contrary, it was displayed that the reduced level of PIGF below the cutoff value was associated with an increased probability of developing preeclampsia with odds ratio 20 [95% confidence interval 10.2–39.5]. These results clearly demonstrated that individual factor assay was associated with an increased probability of developing preeclampsia, but the sFlt-1/PlGF ratio was more precise in prediction of the condition.

Assessment of the performance and accuracy of sFlt-1, PlGF, and the sFlt-1/PlGF ratio (at the optimum cutoff value) as a predictive tool for preeclampsia were established in Table 6. The sFlt-1/PlGF ratio demonstrated the best clinical performance and overall accuracy when compared to individual marker assay.

A cutoff value for sFlt-1/PlGF ratio at 24.5 revealed sensitivity of 91.6%, specificity of 86.4%, positive predictive value (PPV) of 69.1%, negative predictive value (NPV) of 96.9%, positive likelihood ratio (PLR) of 6.7, negative likelihood ratio (NLR) of 0.1, and accuracy of 87.7%, whereas a cutoff value of 3198 pg/mL for sFlt-1 revealed sensitivity of 88%, specificity of 83.6%, PPV of 64%, NPV of 95.4%, PLR of 5.4, NLR of 0.14, and accuracy of 84.7%. While a cutoff value of 138 pg/mL for PlGF revealed sensitivity of 85.5%, specificity of 77.2%, PPV of 55.5%, NPV of 94.1%, PLR of 3.8, NLR of 0.19, and accuracy of 79.3%.

## 4. Discussion

In this study, we supported the suggestion that preeclampsia is associated with the disturbed relationship between angiogenic and antiangiogenic factors. We have evaluated sFlt-1 and PlGF concentrations in a midtrimester pregnant women, and it was established that serum sFlt-1 and PlGF levels in women who subsequently developed preeclampsia behaved differently when compared with women who had a normal pregnancy outcome. Midtrimester levels of sFlt-1 (antiangiogenic factor) elevated and the levels of PlGF (angiogenic factor) declined. Furthermore, when we evaluated the sFlt-1/PlGF ratio for the prediction of preeclampsia, we found that it was significantly higher in women who consequently developed preeclampsia than the control group. Our results established that the clinical performance and overall accuracy of the sFlt-1/PlGF ratio were superior to individual assay for preeclampsia prediction. The serum sFlt-1/PlGF ratio (at a cutoff value of 24.5) was more accurate than sFlt-1 (at a cutoff point of 3198 pg/mL) and PlGF (at a cutoff point of 138 pg/mL) to predict preeclampsia at midtrimester with the best odds ratio and the highest sensitivity, specificity, positive and negative predictive values, positive and negative likelihood ratios, and overall accuracy when compared to the individual factor evaluation.

These data had important clinical implications. Application of a reliable midtrimester method to recognize women at high risk of preeclampsia development could be intensely important, since frequent monitoring and referral to a specialized perinatal care center could considerably reduce maternal and fetal morbidity [26, 27]. Moreover, our data displayed significant differences in sFlt-1 and the sFlt-1/PlGF ratio between patients who subsequently developed preterm and term preeclampsia. Hence, the offspring of women destined to develop preterm preeclampsia could benefit from receiving steroids therapy for fetal lung maturity [28].

The pathogenesis of preeclampsia is still mysterious; however, the last decade carried out a plethora of new and curious data, particularly regarding the role of the angiogenic and antiangiogenic balance in the preeclampsia development. Studies stated that preeclampsia features had a shift in angiogenesis and antiangiogenic factors towards a maladaptive placental circulation with markedly elevated circulating sFlt-1 (antiangiogenic factor) levels and declined levels of PlGF (angiogenic factor) [17, 19, 29].

Hypoxia, caused by imperfect placentation, displays a key event in the pathogenesis of preeclampsia. Placental ischemia/hypoxia perhaps generates the altered angiogenic balance and sponsoring for the antiangiogenic state [30, 31]. The upregulation of sFlt-1 release has been described as a result of a hypoxic environment. Soluble fms-like tyrosine kinase-1 is highly conveyed in the first trimester and so reflects low angiogenic activity and thereby impaired placental development. A consequent sequel of the placental impairment might be fetoplacental hypoxia trailed by a strong subsequent angiogenic activity. High levels of sFlt-1 may be a marker of this activity [5]. High sFlt-1 levels that appear later in pregnancy lead to endothelial dysfunction and mediate preeclampsia development [32].

Likewise, PlGF is speculated to be involved in the etiology of preeclampsia, as PlGF might influence the vascular development of the placental bed and regulate villus development and invasion. Reduced PlGF is linked with higher uteroplacental vascular impedance in mid-pregnancy and noticeably high risks for preeclampsia. The significant relations between declining first-trimester levels and high uteroplacental vascular impedance in mid-pregnancy might label early placental insufficiency [33]. Specific binding of sFlt-1 to PlGF has been advocated as a clarification of reduced PlGF in pregnancies with preeclampsia [5]. It is proposed that sFlt-1 is released by the placenta into the maternal circulation and binds maternal PlGF, leading to maternal endothelial dysfunction [6]. Many animal studies are consistent with this model [5, 34].

Initial studies established that elevated sFlt-1 concentrations were associated with preeclampsia, but only up to five to 10 weeks in advance to the onset of preeclampsia [6]. However, other studies presented elevated sFlt-1 in the first and second trimesters as a risk factor for preeclampsia [35]. On the contrary, Kusanovic et al. [36] and Smith et al. [37] found no association between first-trimester sFlt-1 and preeclampsia. They proposed that an explanation for the development of preeclampsia would be a primary unknown trigger resulting in an abnormal placentation with less effect on the sflt-1 concentration in the first and second trimesters.

Recently, several studies had inspected the possible use of angiogenesis-related factors as biomarkers for the prediction of preeclampsia. Studies of sFlt-1 and PlGF in the second trimester showed that high levels of sFlt-1 and low levels of PlGF might predict preeclampsia development [38–43].

The utility of these markers as an aid in prognostication and diagnosis of preeclampsia was especially useful when a ratio was calculated. The present study showed that the sFlt-1/PlGF ratio improved the sensitivity to predict preeclampsia risk than any of the individual factors. Women with levels of sFlt-1/PlGF ratio greater than 24.5 were at a higher risk of consequent development of preeclampsia compared with women with values lower than that cutoff value. These data were in constant with previous studies which declared that the sFlt-1/PlGF ratio was the best predictor of preeclampsia when compared with individual measure [12, 13]. Levine et al. [6] were the first to provide suggestion that the sFlt-1/PlGF ratio was able to predict the later onset of the disease in women at risk for developing preeclampsia. Also Kusanovic et al. [36] evaluated 1622 singleton pregnancies and they investigated the use of a ratio of sFlt-1 and PlGF. The PlGF/sFlt-1 ratio proved to be a trustworthy tool for the midtrimester prediction of preeclampsia.

## 5. Conclusion

In conclusion, the results offered in the present study for evaluation of sFlt-1 and PlGF concentrations in midtrimester settled and supported the concept that an imbalance in circulating angiogenic and antiangiogenic factors was concomitant with the potential of preeclampsia development and that variation in circulating levels of these particular markers (sFlt-1 and PlGF) preceded the commencement of clinical features of the disease. Moreover, measurement of the sFlt-1/PlGF ratio at midtrimester had a high discriminatory power and overall accuracy to herald the risk of developing preeclampsia. However, this study was limited by being a case-control design in the second trimester and inclusion of patients only from Arabic population. Thus, large prospective multicenter studies including ethnically different populations may be necessary to confirm these results and to employ their clinical practice besides the assessment of the cost-effectiveness of management strategies based on sFlt-1/PlGF ratio testing. As well, we suggest investigating the predictive ability of sFlt-1/PlGF ratio for preeclampsia complications as eclampsia and HELLP syndrome (hemolysis, elevated liver enzymes, and low platelets).

## Figures and Tables

**Table 1:** Demographic and clinical characteristics of women with normotensive pregnancies and women who eventually developed preeclampsia.

Variable	Preeclampsia patients (*n* = 83)	Matched controls (*n* = 250)	*P* value
Characteristics at sampling			
Age (year)	26 (9)	26.5 (11)	0.466
Body mass index (kg/m^2^)	24.7 ± 3.1	23.9 ± 4.6	0.141
Nulliparous, *n* (%)	66 (79.5)	176 (70.4)	0.119
Gestational age (week)	16.9 ± 1.9	16.4 ± 2.8	0.131
Systolic blood pressure (mm Hg)	115.2 ± 14.9	117.1 ± 16.3	0.348
Diastolic blood pressure (mm Hg)	68.6 ± 9.4	70.6 ± 11.6	0.156
Urine protein (dipstick)	Negative*	Negative*	
Characteristics at delivery			
Gestational age at delivery (week)	36.5 ± 1.8	39.2 ± 1.3	<0.0001
Birth weight (g)	2510 (650)	3000 (380)	<0.0001

*Negative, by dipstick analysis.

Data are presented as mean ± standard deviation, median (interquartile range), or number (%).

*P* value < 0.05 is significant.

**Table 2:** Maternal plasma concentrations of sFlt-1, PlGF, and sFlt-1/PlGF ratio in women who developed preeclampsia and in controls.

Variable	Preeclampsia patients (*n* = 80)	Matched controls (*n* = 80)	*P* value
sFlt-1 (pg/mL)	4490 (1080)	2590 (419)	<0.0001
PlGF (pg/mL)	98 (35)	186 (52)	<0.0001
sFlt-1/PlGF ratio	44.6 (16.2)	14.4 (6.1)	<0.0001

sFlt-1: soluble fms-like tyrosine kinase 1; PlGF: placental growth factor.

Data are presented as median (interquartile range).

*P* value < 0.05 is significant.

**Table 3:** Comparison of sFlt-1, PlGF, and sFlt-1/PlGF ratio in term and preterm preeclampsia.

Variable	Term preeclampsia (*n* = 31)	Preterm preeclampsia (*n* = 44)	*P* value
sFlt-1 (pg/mL)	3960 (1268)	4910 (885)	0.01
PlGF (pg/mL)	101 (51)	95 (24)	0.12
sFlt-1/PlGF ratio	35.9 (14.2)	49.5 (12.8)	0.003

sFlt-1: soluble fms-like tyrosine kinase 1; PlGF: placental growth factor.

Data are presented as median (interquartile range).

*P* value < 0.05 is significant.

**Table 4:** Area under the curve from the receiver operating characteristic curve analysis of sFlt-1, PlGF, and sFlt-1/PlGF ratio.

Variable	AUC	95% confidence interval	Sensitivity (%) at FPR		
			5%	10%	15%
sFlt-1	0.875	0.823–0.926	44.6	73.5	83.6
PlGF	0.855	0.81–0.90	14.7	64	74.7
sFlt-1/PlGF ratio	0.917	0.88–0.954	59	81.9	91.6

AUC: area under the curve; FPR: false positive rate; sFlt-1: soluble fms-like tyrosine kinase 1; PlGF: placental growth factor.

False positive rate was calculated as 100 − specificity.

**Table 5:** Optimum cutoff values and odds ratio for preeclampsia according to cutoff values.

Variable	Optimum cutoff value	Odds Ratio	95% Confidence Interval
sFlt-1 (pg/mL)	3198	37.2	17.7–78.1
PlGF (pg/mL)	138	20	10.2–39.5
sFlt-1/PlGF ratio	24.5	67	29.3–162.1

sFlt-1: soluble fms-like tyrosine kinase 1; PlGF: placental growth factor.

**Table 6:** Sensitivity, specificity, PPV, NPV, PLH ratio, NLH ratio, and accuracy of sFlt-1, PlGF, and sFlt-1/PlGF ratio at the optimum cutoff value for prediction of preeclampsia.

Variables	sFlt-1	PlGF	sFlt-1/PlGF ratio
Sensitivity (95% CI)	88 [78.5–93.8]	85.5 [75.7–92]	91.6 [82.9–96.3]
Specificity (95% CI)	83.6 [78.3–87.8]	77.2 [71.4–82.1]	86.4 [81.4–90.3]
PPV (95% CI)	64 [54.5–72.7]	55.5 [46.4–64.2]	69.1 [59.5–77.4]
NPV (95% CI)	95.4 [91.5–97.7]	94.1 [89.8–96.8]	96.9 [93.4–98.6]
PLH ratio (95% CI)	5.4 [4–7.2]	3.8 [2.9–4.8]	6.7 [4.9–9.3]
NLH ratio (95% CI)	0.14 [0.08–0.26]	0.19 [0.11–0.32]	0.1 [0.05–0.2]
Accuracy (%)	84.7	79.3	87.7

sFlt-1: soluble fms-like tyrosine kinase 1; PlGF: placental growth factor; CI: confidence interval; PPV: positive predictive value; NPV: negative predictive value; PLH: positive likelihood ratio; NLH: negative likelihood ratio.
